# Effects of finish rolling temperature and yield ratio on variations in yield strength after pipe-forming of API-X65 line-pipe steels

**DOI:** 10.1038/s41598-020-71729-w

**Published:** 2020-09-08

**Authors:** Dae Woong Kim, Wan-Keun Kim, Jin-ho Bae, Won-Doo Choi, Seok Su Sohn, Sunghak Lee

**Affiliations:** 1 Center for Advanced Aerospace Materials, Pohang University of Science and Technology, Pohang, 37673 Republic of Korea; 2 Steel Products Research Group 1, Pohang Iron and Steel, Gwangyang, 57807 Republic of Korea; 3 Department of Advanced Materials Engineering, Hanbat National University, Daejeon, 305-719 Republic of Korea; 4 Department of Materials Science and Engineering, Korea University, Seoul, 02841 Republic of Korea

**Keywords:** Mechanical properties, Metals and alloys

## Abstract

Flattened plates often show the lower or higher yield strength than initial leveled plates because tensile and compressive strains are repeatedly experienced at outer and inner walls during the pipe-forming and flattening, but reasons for the yield-strength variation after the pipe-forming are not sufficiently verified yet. In this study, ten line-pipe steels were fabricated by controlling alloying elements and finish rolling temperatures (FRTs), and the yield strength of pipe-flattened steel plates was predicted by using cyclic simulation tests, based on competing contributions of Bauschinger effect (BE) and strain hardening (SH) effect quantified from yield drop (YD) and yield rise (YR) parameters, respectively. High-FRT-treated steels (H steels) showed the lower BE and the higher SH than low-FRT-treated steels (L steels), thereby resulting in the smaller yield-strength reduction. This lower BE in the H steels was caused by the lower total boundary density, while the higher SH was caused by the higher fraction of granular bainite. According to the SH analyses between the YR parameters obtained from cyclic simulation tests and the yield ratios obtained from ordinary tensile tests, the decrease in yield-strength reduction with decreasing yield ratio was not attributed to the increase in ordinary tensile SH but to the increase in YR parameter.

## Introduction

Line-pipes used for transportation of natural gas or crude oil have been manufactured by various processes of hot rolling, coiling, leveling, and pipe making, and their final yield strength is measured after pipes are flattened. During these processes, tensile and compressive strains are repeatedly experienced at outer and inner walls, respectively^1–3^. According to such repeated strains, the pipe-flattened plates often show lower or higher yield strength than the leveled plates, which might not satisfy the American Petroleum Institute (API) standards. Thus, large variations in yield strength should be prevented or minimized.

Under repeated piping strains, the reduction in yield strength is generally interpreted by a Bauschinger effect due to back stresses formed by increased mobile dislocations^4,5^. Line-pipe steels generally consist of polygonal ferrite (PF) together with low-temperature-transformed microstructures of granular bainite (GB), bainitic ferrite (BF), and acicular ferrite (AF), and their volume fractions vary with alloying elements and manufacturing thermo-mechanical controlled process (TMCP) parameters^6–10^. Thus, microstructural and processing effects on yield-strength variation should be investigated by carefully considering both Bauschinger effect (BE) and strain hardening (SH). A thickness/diameter ratio (t/D) is generally regarded as a piping strain in piping industries, and affects the yield-strength variation. Thus, effects of microstructural and processing on yield-strength variation should be verified by considering competing mechanisms for the piping, but only a little information is available^2,11,12^.

In this study, line-pipe steels were fabricated by controlling their compositions and finish rolling temperatures (FRTs), and the BE and SH were quantitatively evaluated by yield rise and yield drop parameters, respectively, obtained from cyclic simulation tests to predict the pipe’s yield strength without performing actual piping processes. The variations of yield strength were utilized to understand the processing and microstructural effects of the line-pipe steels. Effect of the decrease in yield ratio, which provides a useful parameter raising the SH obtained from ordinary tensile tests^13,14^, was also discussed.

## Results and discussion

### Microstructures and tensile properties of API-X65 line-pipe steels

**Figure 1:**
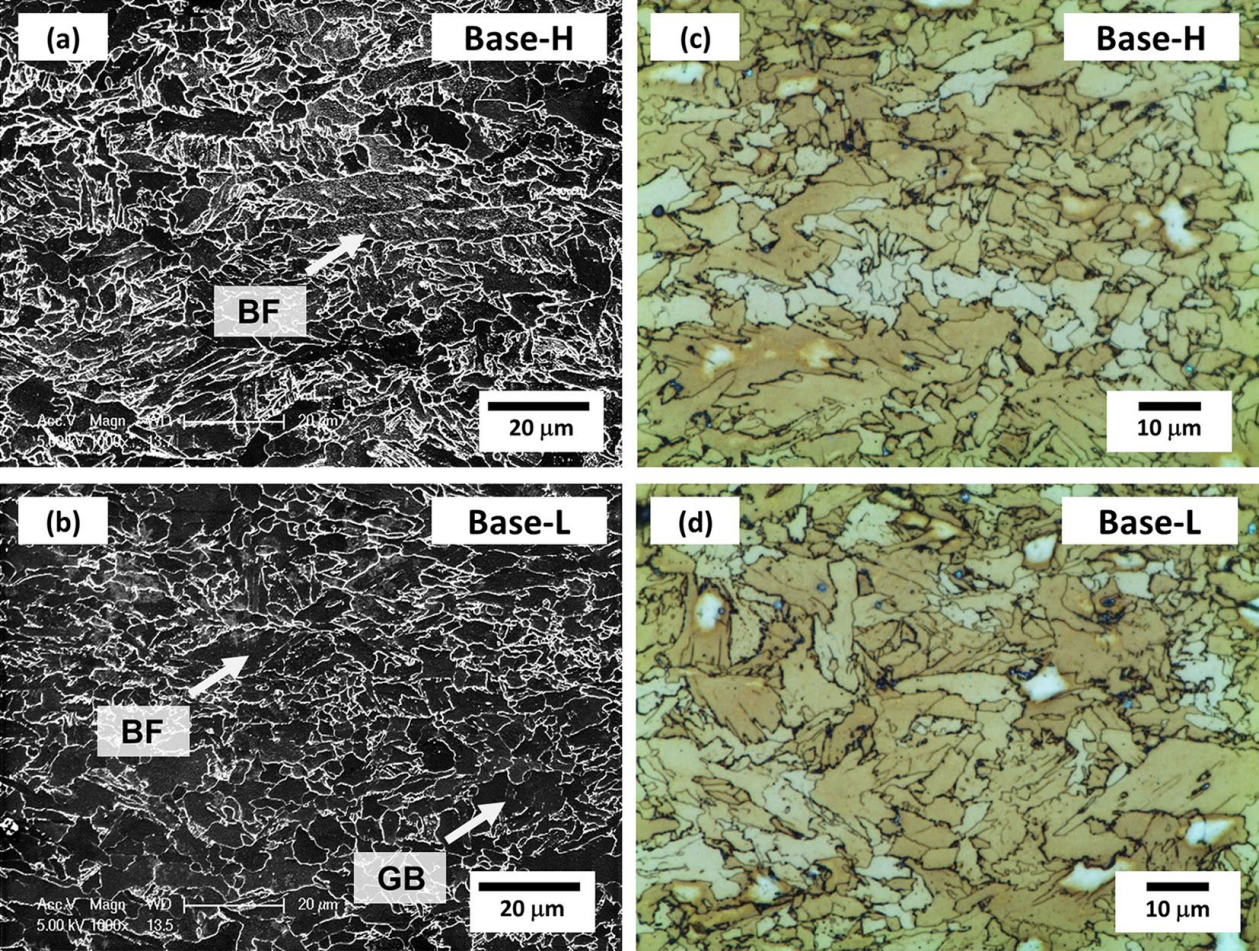
SEM and optical micrographs of the (**a**,**c**) Base-H and (**b**,**d**) Base-L steels. Main microstructures are polygonal ferrite (PF) and granular bainite (GB), along with a small amount of bainitic ferrite (BF) (arrow marks).

Figure 1a–d shows optical and SEM micrographs of the Base-H and Base-L steels. Main microstructures of the two steels are PF and GB (Fig. 1a,b), while a small amount of BF is found (arrow marks). Any MAs are not observed in the LePera-etched optical micrographs (Fig. 1c,d).

**Figure 2:**
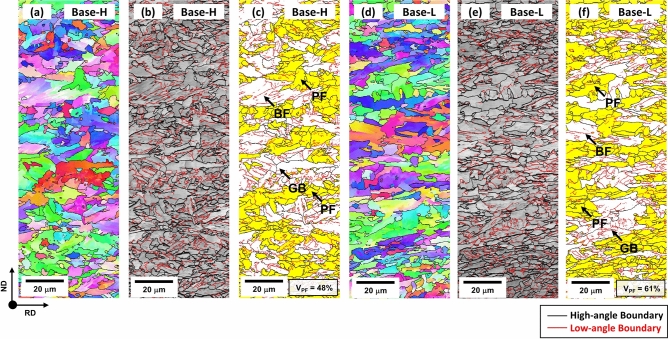
EBSD inverse pole figure (IPF), image quality (IQ), and grain orientation spread (GOS) maps for the (**a**–**c**) Base-H and (**d**–**f**) Base-L steels. High-angle boundaries are differentiated by a criterion of 15 deg in the GOS maps. The PF is regarded as grains of 3 deg in misorientation^15^, as colored in yellow.

Figure 2a–f shows EBSD inverse pole figure (IPF), image quality (IQ), and grain orientation spread (GOS) maps for the Base-H and Base-L steels. Both steels consist mainly of substructure-containing coarse microstructures and randomly-oriented fine microstructures, as shown in Fig. 2a,b,d,e. High-angle boundaries are differentiated by a criterion of 15 deg in the GOS maps (Fig. 2c,f). The PF is regarded as grains of 3 deg in misorientation^15^, as colored in yellow, and is differentiated from coarse-grained GB and BF containing well-developed substructures. The volume fraction of PF is 48 and 61% in the Base-H and Base-L steels, respectively.

**Figure 3:**
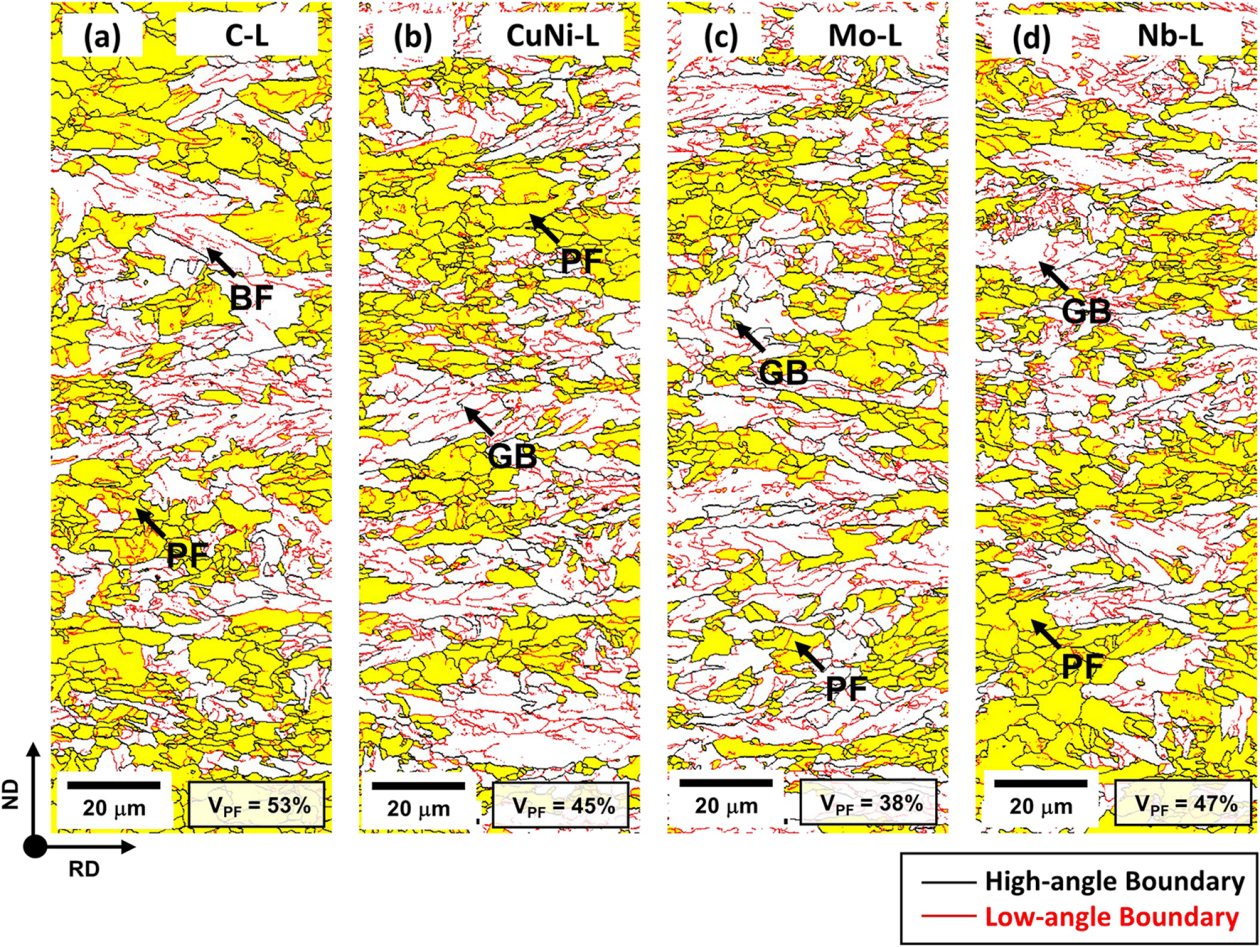
EBSD GOS maps for the (**a**) C-L, (**b**) CuNi-L, (**c**) Mo-L, and (**d**) Nb-L steels. The measured volume fraction of PF (V_PF_) is indicated inside the maps.

By using this microstructural classification technique, major microstructures are also defined in the other steels, and the representative results are shown in Fig. 3a–d. Volume fractions of various microstructures were measured from both micrographs and EBSD maps, and are summarized in Table 1. The Base-H steel contains 48% of PF, 47% of GB, and 5% of BF. The Base-L steel shows the higher PF fraction (61%) and lower GB fraction (36%) than the Base-H steel. When C, Cu + Ni, or Mo is added or Nb is reduced, the fraction of PF decreases, while that of GB increases. When the FRT is compared, the higher FRT causes the lower fraction of PF in all five steel. In the CuNi-H and Mo-H steels, 3 to 5% of AF is also observed. The grain size and total boundary density were measured from EBSD maps, and the results are also listed in Table 1. Here, the total boundary density is defined as the low- and high-angle boundary point number divided by the whole indexed point number^16,17^. In all the steels, the higher FRT causes the larger grain size and the lower boundary density as the fraction of coarse GB rises with increasing FRT.

**Table 1:** Volume fractions of polygonal ferrite (PF), acicular ferrite (AF), granular bainite (GB), and bainitic ferrite (BF), average grain size, and total boundary density in the Base-H, Base-L, C-H, C-L, CuNi-H, CuNi-L, Mo-H, Mo-L, Nb-H, and Nb-L steels (unit: vol%).

Steel	PF	AF	GB	BF	MA	Grain size (μm)	Total boundary density
Base-H	48 ± 2	–	47 ± 3	5 ± 2	–	13.0 ± 0.3	0.253 ± 0.006
Base-L	61 ± 3	–	36 ± 2	3 ± 1	–	10.7 ± 0.6	0.263 ± 0.011
C-H	47 ± 4	–	48 ± 4	5 ± 2	–	14.3 ± 0.5	0.246 ± 0.005
C-L	53 ± 1	–	42 ± 2	5 ± 2	–	12.9 ± 0.5	0.258 ± 0.005
CuNi-H	34 ± 5	3 ± 1	58 ± 4	5 ± 1	–	13.9 ± 0.6	0.285 ± 0.008
CuNi-L	45 ± 2	–	50 ± 3	5 ± 2	–	12.6 ± 0.7	0.295 ± 0.014
Mo-H	31 ± 4	5 ± 2	59 ± 1	5 ± 1	–	17.5 ± 0.9	0.266 ± 0.026
Mo-L	38 ± 2	–	57 ± 2	5 ± 1	–	14.4 ± 0.7	0.300 ± 0.018
Nb-H	37 ± 6	–	60 ± 4	3 ± 1	–	15.9 ± 0.5	0.261 ± 0.009
Nb-L	47 ± 4	–	50 ± 2	3 ± 1	–	12.5 ± 0.3	0.277 ± 0.006

**Figure 4:**
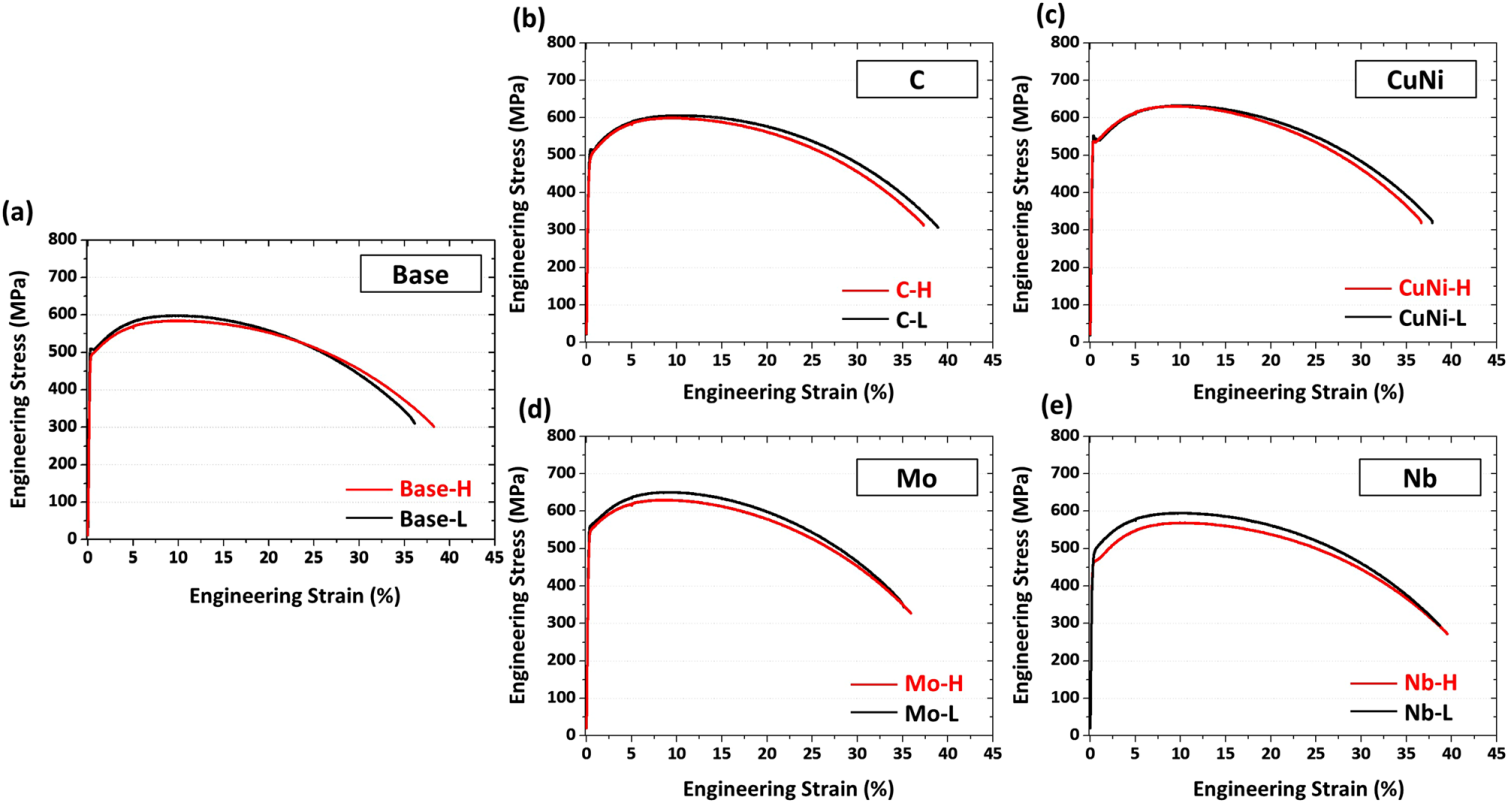
Engineering tensile stress–strain curves of the (**a**) Base, (**b**) C, (**c**) CuNi, (**d**) Mo, and (**e**) Nb steels. All the steels satisfy the minimum yield strength grade of 448 MPa. (Plot: Origin 8, https://www.originlab.com/).

Figure 4a–e shows engineering tensile stress–strain curves of the ten steels at room temperature. The yield strength, tensile strength, and elongation were measured from the stress–strain curves, and are summarized in Table 2. All the steels satisfy the minimum yield strength grade of 448 MPa, and their curve shapes are similar. Yield point phenomenon appears in the Base-L, C-L, CuNi-H, Mo-L and Nb-H steels. The higher FRT causes the lower yield and tensile strengths, while the elongation is similar.

**Table 2:** Room-temperature tensile properties, YR parameters (at 2% pre-strain) and YD parameters of the Base-H, Base-L, C-H, C-L, CuNi-H, CuNi-L, Mo-H, Mo-L, Nb-H, and Nb-L steels.

Steel	Yield strength (MPa)	Tensile strength (MPa)	Total elongation (%)	YR parameter at Pre-strain of 2%	YD parameter
Base-H	494 ± 3	585 ± 2	38.3 ± 0.5	0.093	0.154
Base-L	517 ± 4	599 ± 3	36.1 ± 0.5	0.068	0.169
C-H	503 ± 2	600 ± 5	37.3 ± 0.8	0.076	0.136
C-L	514 ± 4	607 ± 4	38.9 ± 0.5	0.067	0.154
CuNi-H	533 ± 5	632 ± 7	36.6 ± 1.1	0.077	0.152
CuNi-L	545 ± 2	633 ± 5	37.9 ± 0.9	0.063	0.165
Mo-H	564 ± 5	630 ± 2	35.9 ± 0.3	0.076	0.176
Mo-L	562 ± 6	651 ± 3	35.1 ± 0.9	0.065	0.187
Nb-H	483 ± 2	569 ± 6	39.5 ± 1.2	0.096	0.128
Nb-L	495 ± 3	596 ± 3	39.4 ± 1.4	0.084	0.142

### Yield drop and rise parameters of API-X65 line-pipe steels (YD and YR parameters)

**Figure 5:**
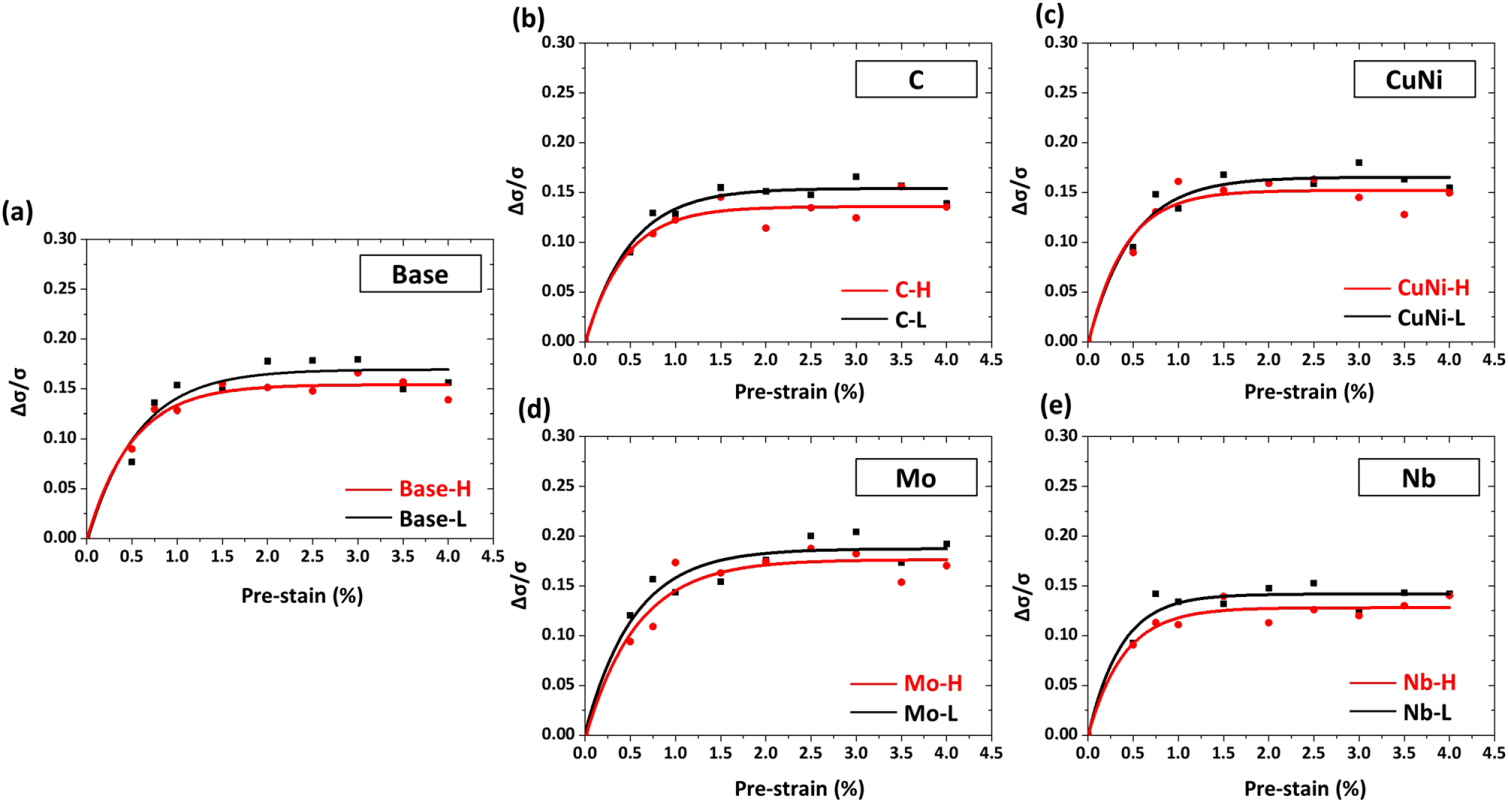
Yield drop (YD) parameters measured at nine pre-strains (0.5, 0.75, 1.0, 1.25, 1.5, 1.75, 2.0, 3.0, and 4.0%) for the (**a**) Base, (**b**) C, (c) CuNi, (**d**) Mo, and (**e**) Nb steels. The YD saturates to a certain value after it increases rapidly until the pre-strain of about 1%. The curves can be fitted in an equation of *y* = *a – b·c*^*x*^ (*a, b,* and *c*; constants). (Plot: Origin 8, https://www.originlab.com/).

Yield drop (YD) parameters were measured at nine pre-strains for the ten steels, and are plotted in Fig. 5a–e as a function of pre-strain. The YD saturates to a certain value after it increases rapidly until the pre-strain of about 1%. The curves can be fitted in an equation of *y* = *a – b·c*^*x*^ (*a, b,* and *c*; constants). The YD of the high-FRT-treated steels (H steels), whose fraction of GB is higher than that of the low-FRT-treated steels (L steels), is lower in the whole pre-strain range. This indicates that the H steels show the lower BE. This also corresponds to the trend of total boundary density data (Table 1). The BE is caused by back stresses induced from pile-ups of dislocations^18^. Constituent phases and their grain sizes affect the number of pile-up sites mainly including low- or high-angle boundaries. In other words, the boundary density determines the pile-up sites that affect the quantity of back stress. Therefore, the boundary density is defined as the most important factor for the BE.

**Figure 6:**
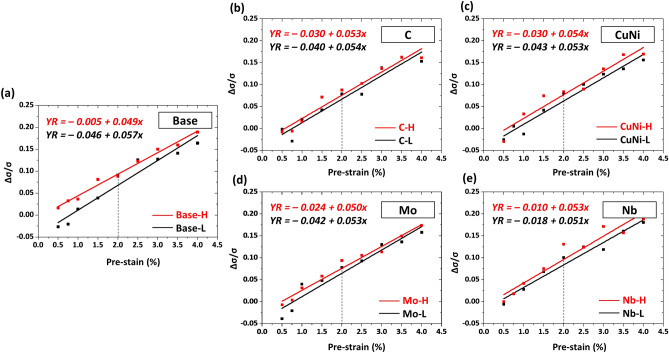
Yield rise (YR) parameters measured at nine pre-strains (0.5, 0.75, 1.0, 1.25, 1.5, 1.75, 2.0, 3.0, and 4.0%) for the (**a**) Base, (**b**) C, (**c**) CuNi, (**d**) Mo, and (**e**) Nb steels. The YR rises linearly with increasing pre-strain in an equation of *y* = *a* + *b·x*, (*a*; constant, *b*; slope). (Plot: Origin 8, https://www.originlab.com/).

Yield rise (YR) parameters are plotted in Fig. 6a–e. The YR rises linearly with increasing pre-strain in an equation of *y* = *a* + *b·x*, (*a*; constant, *b*; slope). In the whole pre-strain range, the YR is higher in the H steels than in the L steel. Though the SH rate of ordinary tensile tests up to the strain of 4% is similar in the H and L steels (Fig. 4a–e), the increase in yield strength is larger in the H steels than in the L steels after the cyclic simulation test. The YR parameter provided in this study is not the factor affected by the SH alone because it is obtained from compression-tension-tension deformation histories. This cyclic deformation also includes the BE. Strain-hardening parameters tend to be high in steels having low BE. Therefore, the SH is largely influenced by the boundary density, which significantly contributes to the BE.

Interestingly, the YD increases rapidly in the low pre-strain range and then saturates (Fig. 5a–e), whereas the YR steadily rises linearly with increasing pre-strain (Fig. 6a–e). This peculiar behavior of YD saturation and linearly-increasing YR is interpreted by the movement of mobile dislocations^5,19,20^ and generation of immobile dislocations^21^, respectively. The behavior of YD saturation phenomenon is consistent with the trend of saturation of mobile dislocations as pre-strain increase^5^. On the other hand, the increasing behavior of YR is related to the increase in immobile dislocation with increasing pre-strain^5^, which is represented as a slope (b) in the equation of YR parameter. Since the YR parameter is also affected by the BE, it is low in steels having a high BE, which is related to a constant (a) in the equation. The YR values are negative because the BE is more dominant than the SH at the low pre-strain range.

It is known that the BE is high in steels where the yield point phenomenon appears^22–24^. In this study, even though the yield-point phenomenon also appears in the Base-L, C-L, CuNi-L, Mo-L, and Nb-H steels, it is difficult to confirm the aforementioned effect. For example, the Base-L steel having the yield-point phenomenon shows the higher BE than the Base-H steel having the continuous yielding behavior. In the same manner, the discontinuous yielding occurs more severely in the Nb-H steel than in the Nb-L steel, but the BE is much lower in the former. In this study, therefore, it can be concluded that changes in BE and SH are affected more by the boundary density than the yielding behavior.

For quantitative analyses of precipitation, an electrochemical extraction method was carried out to estimate volume fractions of hard secondary phases varied with FRT and alloying elements, and contents of Nb, Ti, and Mo existed inside the secondary phases are shown in Table 3. Nb and Ti are detected in all the steels, and Mo is additionally detected in the Mo-L and Mo-H steels. The volume fractions are not significantly varied with the FRT. In this study, thus, the effect of hard secondary phases on BE and SH is hardly explained by the FRT.

**Table 3:** Electrochemical extraction results of the Base-H, Base-L, C-H, C-L, CuNi-H, CuNi-L, Mo-H, Mo-L, Nb-H, and Nb-L steels (unit: wt%).

Steel	Nb	Ti	Mo
Base-H	0.015	0.014	
Base-L	0.013	0.014	
C-H	0.014	0.013	
C-L	0.012	0.013	
CuNi-H	0.012	0.011	
CuNi-L	0.016	0.012	
Mo-H	0.014	0.011	0.005
Mo-L	0.015	0.010	0.005
Nb-H	0.006	0.012	
Nb-L	0.006	0.013	

### Prediction of yield strength of API-X65 line-pipe steels

**Figure 7:**
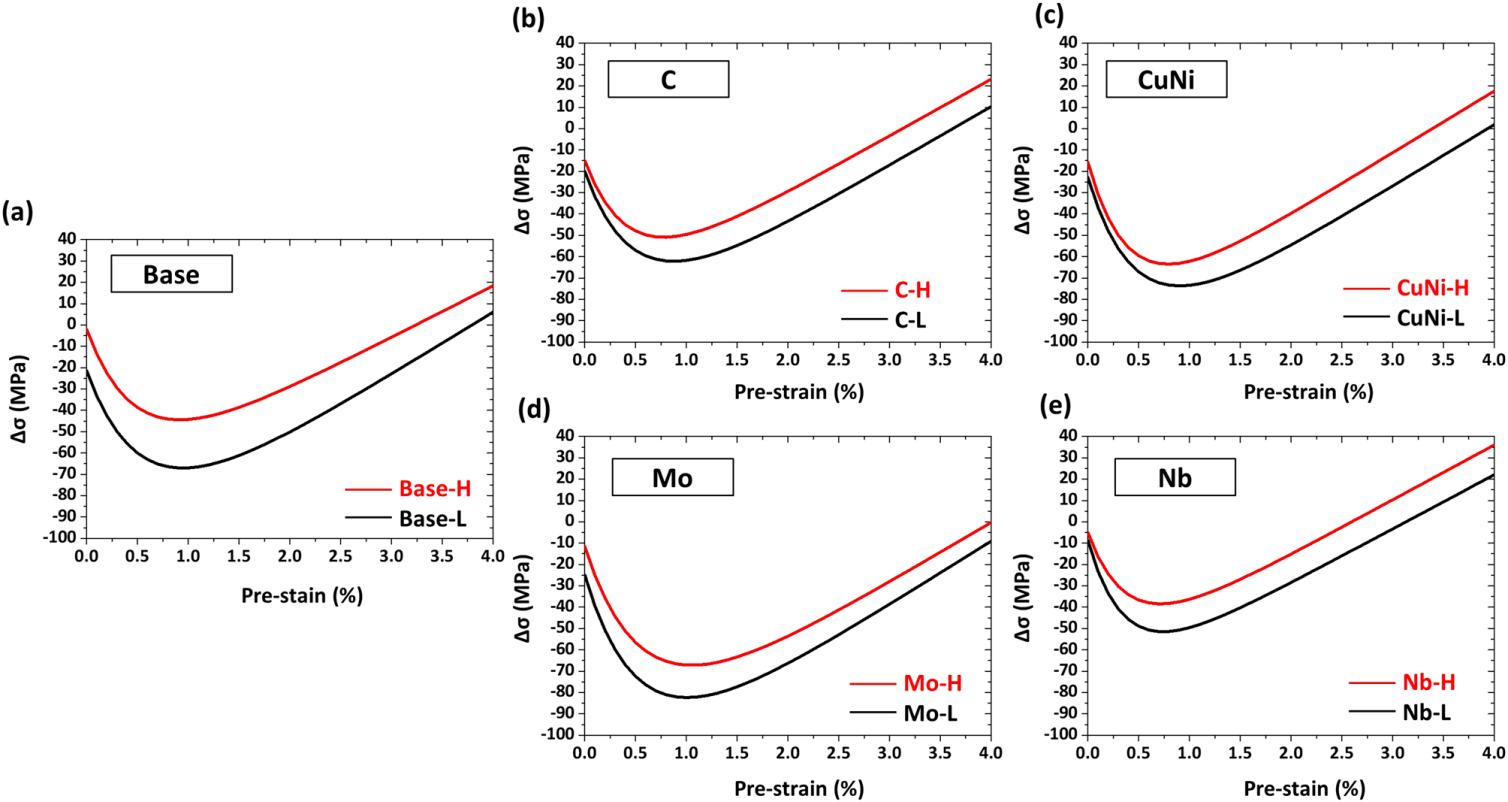
Yield-strength variation (Δσ) obtained by joining YD and YR parameters together for the (**a**) Base, (**b**) C, (**c**) CuNi, (**d**) Mo, and (**d**) Nb steels. The Δσ starts to decrease until the low pre-strain range of 1.0 to 1.5%, and then increases steadily as the BE saturates while the SH steadily rises. (Plot: Origin 8, https://www.originlab.com/).

After the piping, the yield strength can be predicted by simultaneously considering the BE and SH which can be reliably interpreted by YD and YR parameters, respectively. The yield-strength variation (Δσ) was obtained by joining YD and YR parameters together after YD curves are reversed upside down, and the Δσ results of the ten steels are shown in Fig. 7a–e as a function of pre-strain. The Δσ decreases until the low pre-strain range of 1.0 to 1.5%, and then increases steadily as the BE saturates (Fig. 5a–e) while the SH steadily rises (Fig. 6a–e). These down-and-up-shape Δσ curves appear similarly in both the H and L steels. In the L steels, however, the curve decreases more in the low pre-strain range below the initial tensile yield strength of the leveled plate (Δσ = 0) because the BE of the L steels is higher than that of the H steels while the SH is lower.

Since the yield strength of flattened plates is lower or higher than that of leveled plates, much research on accurate prediction and reduction in Δσ has been conducted^25–27^. It has been frequently observed that the BE and SH prevail in low and high piping strain (t/D) or pre-strain range, respectively^25,26^. In linepipe steels, problems of severe drop in yield strength after the pipe forming usually occur between t/D range of 0.01 and 0.02^17^. The drop in yield strength is not severe over t/D of 0.02 because the effect of SH is much greater than the BE. The range of severe drop in yield strength corresponds to 0.5–1.5% of pre-strain range of the present study. Until the pre-strain of 1.5%, the BE increases rapidly, where the SH is relatively weak. The drop in yield strength is not severe because the SH increases gradually over the pre-strain of 1.5%. This trend is well illustrated in Figs. 6 and 7. Although it is difficult to determine the exact range, the pre-strain of 1.5% is regarded as a marginal value. The varying Δσ curve shape in the low t/D range has been recognized in piping industries, but its reason or mechanism has hardly been explained yet. It is challenging to analyze the cause of drop in yield strength in flattened sheets because it is hard to know which factor is dominant between BE and SH. Nevertheless, the separate evaluation of strength variation at inner and outer walls enables to identify the cause. In this study, the Δσ predicted by joining YD and YR parameters together on the assumption that the applied strains at inner and outer walls are identical is reliably explained by the predominance of BE and SH in the low and high t/D ranges, respectively. It is also useful to understand that the high FRT is beneficial for reducing the Δσ because of the lower YD and the higher YR parameters than the low FRT, which provides a useful guidance on processing of line-pipe steels.

### Effects of alloying elements on yield-strength variation of API-X65 line-pipe steels

**Figure 8:**
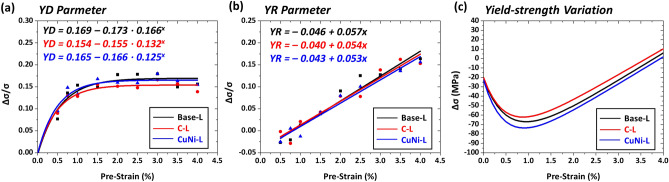
(**a**) Yield drop (YD) parameter, (**b**) yield rise (YR) parameter measured at nine pre-strains, and (**c**) yield-strength variation (Δσ) as a function of pre-strain for the Base-L, C-L, and CuNi-L steels. The yield-strength reduction (− Δσ) is largest in the CuNi-L steel throughout the pre-strain range, and is smallest in the C-L steel. (Plot: Origin 8, https://www.originlab.com/).

Effects of solid-solution-hardening elements such as C and Cu + Ni on Δσ were investigated by comparing the BE and SH in the Base-L, C-L, and CuNi-L steels. Figure 8a–c shows variations in YD and YR parameters and yield strength as a function of pre-strain. The overall BE expressed by YD parameters decreases as C is added to the Base steel, and is hardly varied with the Cu + Ni addition (Fig. 8a), whereas the SH expressed by YR parameters is similar in the three steels (Fig. 8b). When the YD and YR parameters are combined to estimate the Δσ, the yield-strength reduction (− Δσ) is largest in the CuNi-L steel throughout the pre-strain range, and is smallest in the C-L steel. The smallest − Δσ in the C-L steel is attributed to the reduced BE, while the SH is similar to the other steels. The larger − Δσ of the CuNi-L steel than the Base steel (Fig. 8c) in spite of the similar YD and YR parameters (Fig. 8a,b) is caused by the higher initial tensile yield strength than the Base-L steel (545 *vs*. 517 MPa, Table 2). Thus, the solid-solution hardening effect of C works favorably for decreasing the − Δσ, although that of Cu + Ni does not work.

**Figure 9:**
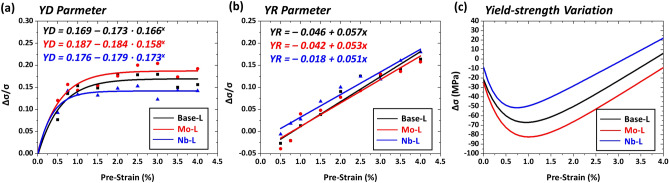
(**a**) Yield drop (YD) parameter, (**b**) yield rise (YR) parameter measured at nine pre-strains, and (**c**) yield-strength variation (Δσ) as a function of pre-strain for the Base-L, Mo-L, and Nb-L steels. The yield-strength reduction (− Δσ) is largest in the Mo-L steel throughout the pre-strain range because the BE is highest while the SH is lowest, and decreases in the order of the Base-L and Nb-L steels. (Plot: Origin 8, https://www.originlab.com/).

Effects of precipitation-hardening elements such as Mo and Nb on Δσ were investigated on the Base-L, Mo-L, and Nb-L steels, and the results of YD, YR, and Δσ are shown in Fig. 9a–c. The quantitative precipitation data are shown in Table 3. The overall BE increases in the order of the Nb-L, Base-L, and Mo-L steels (Fig. 9a), while this order is reversed in the overall SH (Fig. 9b). As a result, the − Δσ is largest in the Mo-L steel throughout the pre-strain range because the BE is highest while the SH is lowest, and decreases in the order of the Base-L and Nb-L steels, as shown in Fig. 9c. This result indicates that the addition of Mo shows the largest − Δσ, whereas the reduction in Nb induces the smallest − Δσ. The addition of Mo and Nb promotes the precipitation of fine Mo and Nb carbo-nitrides^28–30^, as shown in the Table 3. This precipitation usually generates back stresses by the interaction of carbo-nitrides and dislocations to readily raise the BE^31–33^. Thus, the precipitation hardening elements of Mo and Nb work unfavorably for decreasing the − Δσ.

### Effects of yield ratio on yield-strength variation

A decrease of yield ratio (or an increase in SH rate) measured from ordinary tensile tests of line-pipe steels has been suggested by the piping researchers to effectively reduce the yield strength decrease after the piping^11,34^. In fact, the reduction of the yield strength change has been observed as the yield ratio decreases^11,34^, and thus the decrease in yield ratio is recognized as a dominant method for increasing the SH even during the piping. However, this idea is not clear because overall stress and strain states are basically different in compression-tension-tension cyclic simulation and ordinary tensile tests^17^. As a result, the SH related with the YR parameter obtained from cyclic simulation tests for the piping might be different from that related with the yield ratio obtained from ordinary tensile tests.

**Figure 10:**
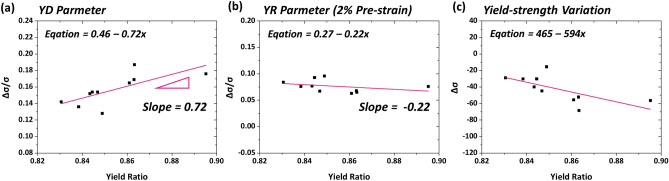
(**a**) YD parameter, (**b**) YR parameter (at 2% pre-strain), and (**c**) yield-strength variation (Δσ) as a function of yield ratio for the present 10 steels. The YD parameters tend to increase continuously with increasing yield ratio, whereas the YR parameters are hardly varied. The yield-strength reduction (− Δσ) increases steadily with increasing yield ratio because the YD parameter increases while the YR parameter remains. (Plot: Origin 8, https://www.originlab.com/).

The SH relation between the yield ratio and YR parameter is statistically investigated by using the microstructural and mechanical-test data of Tables 1 and 2. Figure 10a,b shows plots of YD and YR parameters of the present ten steels as a function of yield ratio. Here, the YD parameters are collected from the saturation values in Fig. 5a–e, which are hardly varied in a relatively high pre-strain range, and are summarized in Table 2. The YR parameters, which linearly increase as the pre-strain increases, are collected from the pre-strains of 2%, as indicated by dashed lines in Fig. 6a–e. The YD parameters tend to increase continuously with increasing yield ratio, whereas the YR parameters are hardly varied. By combining these YD and YR parameters, the Δσ is plotted as a function of yield ratio, as shown in Fig. 10c. The yield-strength reduction (− Δσ) increases steadily with increasing yield ratio because the YD parameter increases (Fig. 10a) while the YR parameter remains (Fig. 10b). This tendency corresponds well with the reduction in -Δσ with decreasing yield ratio, as expected in piping industries, but the main reason for this is not simply explained by the decrease in yield ratio due to the high SH. This is because the SH effect (represented by YR) is different from that in engineering tensile stress–strain curves (represented by yield ratio). That is, the decrease in -Δσ with decreasing yield ratio is not attributed to the increase in ordinary tensile SH but to the decrease in YD parameter obtained from cyclic simulation tests. These comparison results of yield strength provide a promising method for interpreting SH effects on Δσ by combining both BE and SH occurring during the piping.

## Conclusions

In this study, ten line-pipe steels were fabricated by controlling alloying elements and finish rolling temperatures (FRTs), and the yield strength of their pipes was predicted, based on competing contributions of Bauschinger effect (BE) and strain hardening (SH) quantified from cyclic simulation tests.

1. After the piping, the yield strength could be predicted by considering both BE and SH which were reliably interpreted by yield drop (YD) and yield rise (YR) parameters, respectively. The yield-strength variation (Δσ) was obtained by combining YD and YR parameters after the YD curves were reversed upside down. The yield strength variation first decreased until the low pre-strain range of 1.0 to 1.5%, and then increased as the BE saturated while the SH steadily increased.
2. According to the YD curve analyses, high-FRT-treated steels (H steels) showed the lower BE and the higher SH than low-FRT-treated steels (L steels), thereby resulting in the smaller decrease in yield strength. The lower BE in the H steels corresponded to the trend of total boundary density because the BE increased with increasing boundary density as the back stress and BE were effectively raised by many grain boundaries.
3. Effects of solid-solution-hardening elements (C and Cu + Ni) and precipitation-hardening elements (Mo and Nb) on Δσ were investigated by comparing the BE and SH expressed by YD and YR parameters, respectively. The solid-solution hardening effect of C worked favorably for decreasing the yield-strength reduction (− Δσ), although the (Cu + Ni) addition did not work. The precipitation hardening elements of Mo and Nb worked unfavorably for decreasing the − Δσ because the precipitation of carbo-nitrides generated back stresses to raise the BE.
4. The SH analyses between the YR parameter obtained from cyclic simulation tests and the yield ratio obtained from ordinary tensile tests were statistically investigated. The YD parameters tended to continuously increase with increasing yield ratio, whereas the YR parameters were hardly varied. When combining these YD and YR parameters, the yield-strength reduction (− Δσ) increased steadily with increasing yield ratio because the YD parameter increased while the YR parameter remained. Thus, the decrease in − Δσ with decreasing yield ratio was not attributed to the increase in ordinary tensile SH but to the decrease in YD parameter.

## Method

### Fabrication of line-pipe steels

API X65-grade (minimum yield strength; 448 MPa) line-pipe steels were fabricated by a vacuum-induction melting, and the chemical compositions are listed in Table 4. The steel having a basic composition of (< 0.07)C–(< 0.3)Si–(< 1.5)Mn–(< 0.02)Ti + V–(< 0.05)Nb (wt%) is referred to as ‘Base’, and alloying elements of C, Cu + Ni, Mo, and Nb were added into the reference composition to produce ‘C’, ‘CuNi’, ‘Mo’, and ‘Nb’ steels, respectively. 90-mm-thick plates were homogenized at 1523 K for 1 h and hot-rolled at 1,373–1,073 K to produce 12-mm-thick plates. They were cooled to 813 to 843 K from finish rolling temperatures of 1,133–1,143 K or 1,073–1,083 K (above Ar_3_), held at this temperature for 1 h (for the coiling simulation), and then cooled to room temperature. For convenience, the steel plates finish-rolled at high and low temperatures (1,133–1,143 K and 1,073–1,083 K, respectively) are referred to as ‘H’ and ‘L’, respectively.

**Table 4:** Nominal chemical compositions of the Base, C, CuNi, Mo, and Nb steels (unit: wt%).

Steel	C	Si	Mn	Ti + V	Nb	Cu + Ni	Mo
Base	< 0.07	< 0.3	< 1.5	< 0.02	< 0.05	–	–
C	< 0.08					–	–
CuNi	< 0.07					< 0.4	–
Mo	< 0.07					–	< 0.1
Nb	< 0.07				< 0.03	–	–

### Microstructural characterization and tensile testing

Steel plates were polished and etched in a 2% nital solution. The microstructures of longitudinal-short-transverse (L-S) plane were observed by optical and scanning electron microscopes. A LePera-solution-etching^35^ was also adopted for identifying martensite-austenite constituents (MAs). Electron back-scattered diffraction (EBSD) analyses (step size 0.15 μm) were performed on electro-polished specimens. Round tensile bars (gage length: 12.5 mm; diameter: 6.35 mm; orientation: transverse) were tensioned at a strain rate of 5 × 10^−3^ s^−1^ at room temperature by a universal testing machine (8801, Instron, USA, capacity; 100 kN). All tests were carried out three times to ensure the data reliability.

### Cyclic simulation tests

Cyclic simulation tests composed of tensile-compressive or compressive-tensile test followed by tensile test were conducted to simulate piping and flattening procedures. The strain (*ε(X)*) is defined as *ε(X)* = 2*X*/(*D*-*t*), where *D*, *t*, and *X* are outer diameter, thickness, and distance from the plate center, respectively^36^. Considering the strain history varied with the location of plate thickness, nine pre-strains (*ε*_*pre*_, 0.5, 0.75, 1.0, 1.25, 1.5, 1.75, 2.0, 3.0, and 4.0%) were selected.

**Figure 11:**
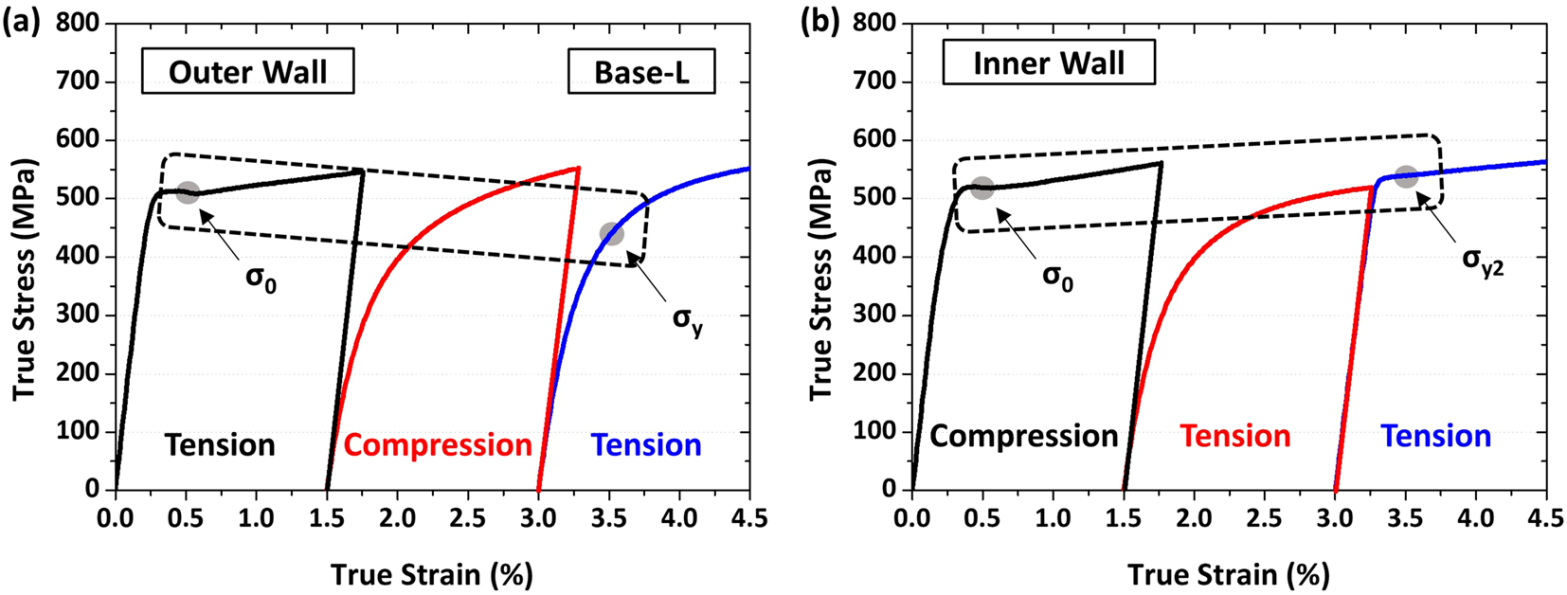
Typical true (**a**) tensile-compressive-tensile and (**b**) compressive-tensile-tensile cyclic test curves of the Base-L steel at the pre-strain of 1.5% to simulate the pipe-forming and flattening processes of the outer and inner walls. Yield drop (YD) and yield rise (YR) parameters are quantified from tensile-compressive-tensile and compressive-tensile-tensile test curves, respectively. (Plot: Origin 8, https://www.originlab.com/).

To simulate the outer wall of the pipe for estimating the yield strength, tensile-compressive-tensile cyclic tests were conducted on the cyclic-simulation test specimen (same to tensile test specimen) under nine pre-strains. A yield drop parameter (YD) is newly defined to be *YD* = *(σ*_0_* –* *σ*_*y*_*)/σ*_0_, where σ_0_ and *σ*_*y*_ are the initial tensile yield strength and the yield strength obtained from the tensile-compressive-tensile cyclic-simulation test, respectively. A schematic drawing of true tensile-compressive-tensile curves of the Base-L steel at the pre-strain of 1.5% is shown in Fig. 11a. This YD is useful for accurately simulating the outer wall via tensile-compressive-tensile cyclic test, instead of the previous YD definition method using the tensile-compressive cyclic test^16,17^. To simulate the inner wall, the specimen was compressive-tensile-tested, and then was tensile-tested for evaluating the yield strength. A yield rise parameter (YR), representing the SH occurring during the piping, is defined to be *YR* = *(σ*_*y*2_* –* σ_0_*)/*σ_0_, where σ_*y*2_ is the final yield strength after the compressive-tensile-tensile cyclic test, as schematically exemplified in Fig. 11b for the inner wall of the Base-L steel. These YD and YR parameters express reasonably the BE and SH occurring during the piping.

## Data Availability

The data that support the findings of this study are available from the corresponding author upon reasonable request.
